# Longitudinal analysis reveals age-related changes in the T cell receptor repertoire of human T cell subsets

**DOI:** 10.1172/JCI158122

**Published:** 2022-09-01

**Authors:** Xiaoping Sun, Thomas Nguyen, Achouak Achour, Annette Ko, Jeffrey Cifello, Chen Ling, Jay Sharma, Toyoko Hiroi, Yongqing Zhang, Chee W. Chia, William Wood, Wells W. Wu, Linda Zukley, Je-Nie Phue, Kevin G. Becker, Rong-Fong Shen, Luigi Ferrucci, Nan-ping Weng

**Affiliations:** 1 Laboratory of Molecular Biology and Immunology,; 2 Gene expression and Genomics Unit, Laboratory of Genetics and Genomics, and; 3 Laboratory of Clinical Investigation, National Institute on Aging, NIH, Baltimore, Maryland, USA.; 4 Facility for Biotechnology Resources, Center for Biologics Evaluation and Research, Food and Drug Administration, Silver Spring, Maryland, USA.; 5 Translational Gerontology Branch, National Institute on Aging, NIH, Baltimore, Maryland, USA.

**Keywords:** Aging, Adaptive immunity

## Abstract

A diverse T cell receptor (TCR) repertoire is essential for protection against a variety of pathogens, and TCR repertoire size is believed to decline with age. However, the precise size of human TCR repertoires, in both total and subsets of T cells, as well as their changes with age, are not fully characterized. We conducted a longitudinal analysis of the human blood TCRα and TCRβ repertoire of CD4^+^ and CD8^+^ T cell subsets using a unique molecular identifier–based (UMI-based) RNA-seq method. Thorough analysis of 1.9 × 10^8^ T cells yielded the lower estimate of TCR repertoire richness in an adult at 3.8 × 10^8^. Alterations of the TCR repertoire with age were observed in all 4 subsets of T cells. The greatest reduction was observed in naive CD8^+^ T cells, while the greatest clonal expansion was in memory CD8^+^ T cells, and the highest increased retention of TCR sequences was in memory CD8^+^ T cells. Our results demonstrated that age-related TCR repertoire attrition is subset specific and more profound for CD8^+^ than CD4^+^ T cells, suggesting that aging has a more profound effect on cytotoxic as opposed to helper T cell functions. This may explain the increased susceptibility of older adults to novel infections.

## Graphical abstract

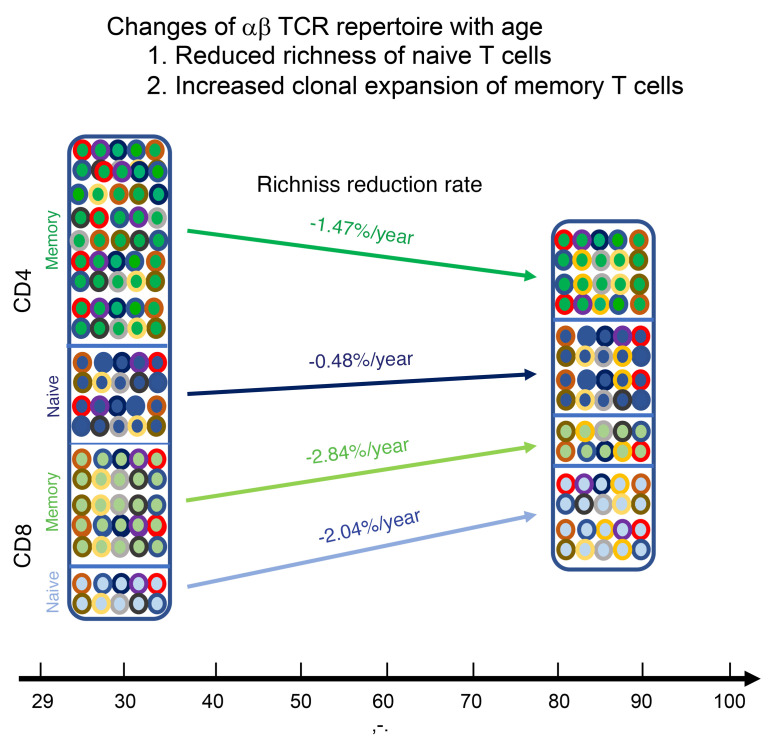

## Introduction

Naive T cells produced by the thymus have the potential to recognize any pathogen, whereas memory T cells are generated from a past immunological response and offer long-lasting protection against pathogens during a subsequent encounter (1–4). Production of naive T cells substantially declines after puberty, creating a challenge to maintaining a T cell system throughout a lifetime that balances the numbers of naive and memory T cells (5, 6). Memory T cells continuously accumulate with various degrees of selective clonal expansion after new or repeated immune responses (7, 8). There are 2 general types of T cells: CD4^+^ cells primarily offer a helper function via the release of cytokines to promote and regulate functions of both B cells (humoral response) and CD8^+^ T cells; and CD8^+^ cells use their cytotoxic pathways to kill virally infected or cancerous cells. With age, the reduction in naive T cells in circulating blood is more severe for CD8^+^ than CD4^+^ T cells, although the rate of naive CD8^+^ T cell loss varies tremendously among healthy adults (9–13). Studying the dynamics of naive and memory CD4^+^ and CD8^+^ T cells throughout the adult lifetime is important for understanding immunity and aging. Details at the level of T cell receptor (TCR) clonotypes are necessary to understand the age-associated changes of TCR repertoire, which is measured by T cell richness, meaning the number of unique TCR sequences in an individual’s T cell repertoire. Currently, the dynamics of naive and memory CD4^+^ and CD8^+^ T cells throughout the adult lifetime at the level of TCR sequence are not known.

The repertoire of αβ TCR — the TCR variable segments that recognize specific pathogens — is determined in adult humans by both genetic events, such as recombination of variable gene regions and α-β chain pairing, and the number of mature T cells in the body. The estimated number of T cells in circulation in an adult is approximately 4 × 10^11^ (14), with estimates of αβ TCR repertoire size based on genetic elements as high as 1 × 10^15^ (15–17). Experimental analyses of TCRβ sequences from small numbers of T cells (~1 × 10^6^) suggest that the predicted TCRβ repertoire size range is 1 × 10^6^–10^8^ (18, 19). In addition, TCRβ repertoires appear to have reduced richness with age for both total T cells (20–22) and naive and memory CD4^+^ and CD8^+^ cells (23). Research shows that TCRβ repertoire size is highly diverse in human adults (20–22) and is substantially different between naive and memory T cells (23–25), but longitudinal analysis of actual TCR repertoire changes in naive and memory T cells with age using human samples are lacking. Furthermore, reports on TCRβ repertoire size as it corresponds with age were performed with the assumption that all study participants had equal number of T cells without considering the substantial individual differences in T cell numbers and their changes with age. Finally, little is known about size and age changes in the TCRα repertoire or the actual αβ TCR repertoire in humans.

Naive T cells are long-lived cells (26), and the main route for maintaining the naive T cell pool throughout adulthood in humans is homeostatic proliferation (5). The survival of naive T cells depends on having received maintenance signals through the TCR as well as having been exposed to cytokines such as IL-7 in lymphoid organs (27, 28). This naive T cell maintenance mode appears unbiased in early adulthood, but selective expansion of certain naive T cell clones is reported in older humans (23, 29). However, when the uneven expansion of naive T cells starts in an adult life and whether this uneven expansion continues or occurs randomly with age are unknown. Cumulative homeostatic proliferation has 2 known consequences: (a) altered activation thresholds of naive T cells to antigenic activation (30, 31), and (b) loss of naive phenotype and gain in memory phenotype, which is not because of differentiation induced by cognate antigen stimulation (32–35). These alterations are largely characterized by their phenotypes and activation-induced response, but the clonal evidence of these changes has not been determined.

In this study, we conducted a longitudinal assessment of the TCRα and TCRβ repertoires in naive and memory CD4^+^ and CD8^+^ T cells from healthy adults. We applied RNA-seq with a TCR-mRNA-marking method using unique molecular identifiers (UMI) to reduce the errors of sequencing read–based methods. We determined longitudinal changes in TCR repertoire and projected TCR repertoire size using the actual circulating T cell numbers from participants’ blood provided at each of 2 donations. We developed equations to calculate αβ TCR repertoire size from TCRα and TCRβ sequences. Our study demonstrated that increasing age is associated with (a) reduced αβ TCR repertoire richness in CD4^+^ and CD8^+^ T cells, particularly in naive CD8^+^ T cells; (b) increased clonal expansion of memory CD8^+^ T cells; (c) increased overlap in TCR sequences in longitudinal samples for both CD4^+^ and CD8^+^ T cells, particularly memory CD8^+^ T cells; and (d) reduced distinction of TCR sequences between naive and memory CD4^+^ and CD8^+^ T cells as well as between CD4^+^ and CD8^+^ T cells. These findings, based on actual T cell numbers in individual healthy adults, reveal the dynamic in vivo changes with age in naive and memory CD4^+^ and CD8^+^ T cells at the resolution of TCRα and TCRβ sequences.

## Results

### Reduction of TCRα and TCRβ repertoires in CD4^+^ and CD8^+^ T cells with age.

**Figure 1:**
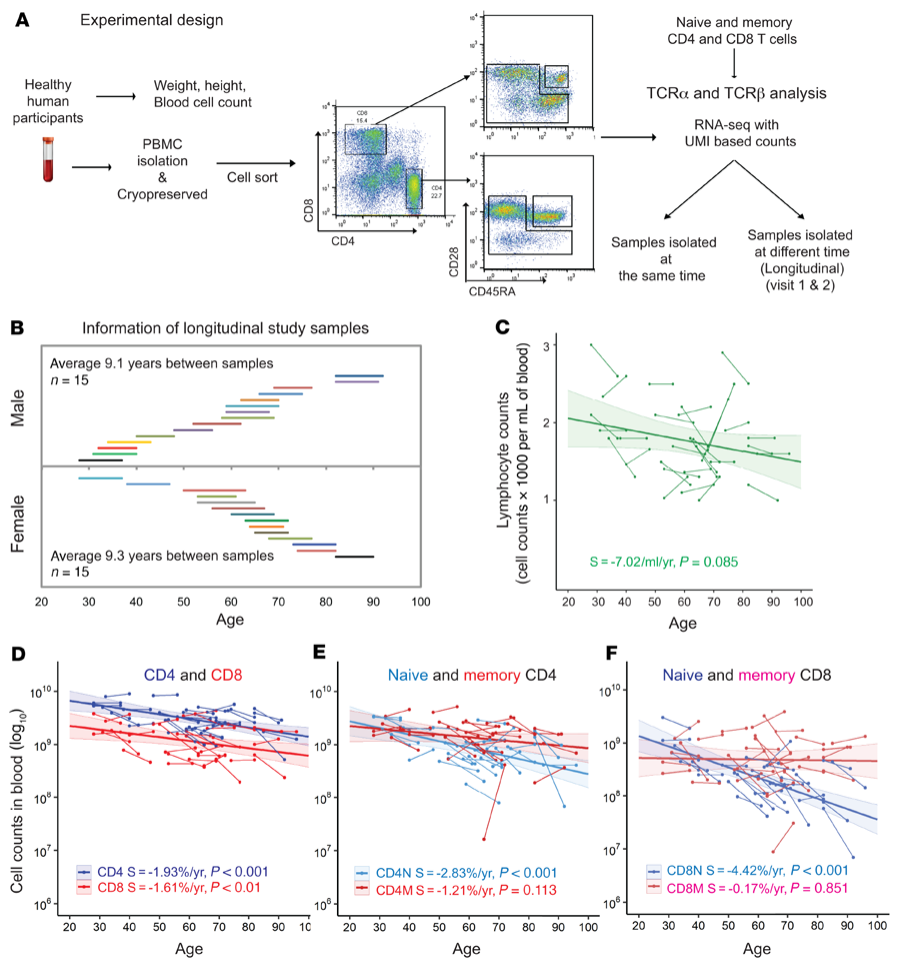
Experimental scheme. (**A**) Experimental design. Thirty healthy adults were selected from participants of the Baltimore Longitudinal Study of Aging (BLSA). At each of 2 visits, weight and height were measured and fasting blood was drawn, and PBMCs were isolated and cryopreserved. From each sample, CBC counts were analyzed. For experiments, PBMCs were thawed and stained for CD4, CD8, CD45RA and CD28. Naive and memory CD4^+^ and CD8^+^ T cells were isolated by cell sorting for T cell receptor α (TCRα) and TCRβ repertoire analysis. PBMCs were isolated from 3 additional healthy adults and naive and memory CD4^+^ and CD8^+^ T cells were sorted in 3–4 aliquots as reproducibility controls. UMI, unique molecular identifier. (**B**) Age and sex of participants at first and second donation. Each line represents 1 donor, and the length of line indicates years between donations. (**C**–**F**) Numbers of lymphocytes, total, naive, and memory CD4^+^ and CD8^+^ T cells in samples from 2 donations, with change with age. Cell numbers were based on (a) lymphocyte counts per microliter of blood; (b) percentage of CD4^+^ and CD8^+^ T cells and naive and memory cells in lymphocytes, calculated from flow cytometry; and (c) blood volume calculated from donor weight and height adjusted by sex (39). Thin short lines link 2 donations from 1 participant. The thick long line is the trend from MLE analysis. The colored shade around the trend line indicates 95% confidence interval. Unless otherwise noted, values were transformed with log_10_ for presentation and statistical analysis. Values for slope (S) of the trend line and *P* values (≤ 0.05 was considered significant) are presented. N, naive; M, memory T cells.

To determine changes in αβ TCR repertoires with age, we isolated CD4^+^ and CD8^+^ T cell subsets from cryopreserved PBMCs of 30 healthy humans. Using samples taken an average of 9.2 years apart, we determined TCRα and TCRβ repertoires using a UMI-based RNA-seq method (Figure 1A and Supplemental Table 1; supplemental material available online with this article; https://doi.org/10.1172/JCI158122DS1) (36–38). The age of study donors at first visit was late 20s to early 80s, with equal numbers of male and female participants (Figure 1B). To accurately measure TCR repertoire changes with age, we first determined the numbers of circulating CD4^+^ and CD8^+^ T cells in the blood of each participant at each donation. The total number of T cells and their subsets in an individual were calculated by (a) determining the percentages of CD4^+^ and CD8^+^ T cells and their subsets by flow cytometry of lymphocytes; (b) calculating the counts of CD4^+^ and CD8^+^ T cells and their subsets in a microliter of blood based on complete blood cell counts (CBCs); (c) determining total blood volume based on donor height and weight at donation using Nadler’s Equation (39); and (d) calculating the number of CD4^+^ and CD8^+^ T cells and their subsets in the donor’s total blood. We observed a significant reduction in numbers of lymphocytes, CD4^+^ and CD8^+^ T cells, and naive CD4^+^ and CD8^+^ T cells with age (Figure 1, C–F, Supplemental Figure 1, and Supplemental Table 1). These actual numbers of T cells in the blood for each donor were used for projected TCR repertoire richness.

**Figure 2:**
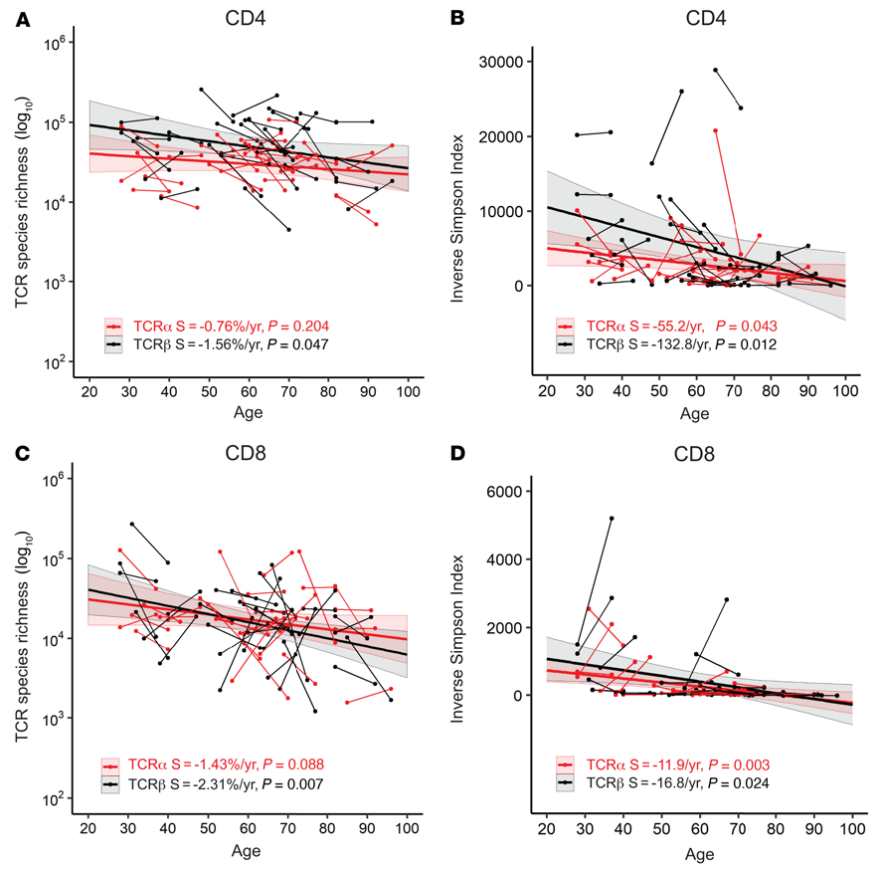
Reductions in αβ TCR repertoires in CD4^+^ and CD8^+^ T cells with age. (**A**) Age-associated reduction in projected richness of T cell receptor α (TCR α) and TCRβ repertoires of CD4^+^ T cells. TCRα and TCRβ sequences were calculated for each donor and projected to 1% of total circulating CD4^+^ T cells (in log_10_-based values) (see **C**). (**B**) Age-associated reduction of TCRα and TCRβ diversity of CD4^+^ T cells measured by ISI. (**C**) Age-associated reduction in projected richness of TCRα and TCRβ repertoires of CD8^+^ T cells. (**D**) Age-associated reduction of TCRα and TCRβ diversity of CD8^+^ T cells measured by ISI. The colored shade around the trend line indicates the 95% confidence interval. S, slope of the trend line.

After analysis of 1.9 × 10^7^ individual TCRα and TCRβ mRNA molecules (UMI counts) from 1.9 × 10^8^ isolated T cells from 30 donors with an average sequencing depth of approximately 30 sequencing reads per UMI, we calculated (a) TCR repertoire richness that measures the number of unique TCRs in a donor by rarefaction equations that project to the actual numbers of circulating T cells in the blood (1% of total T cell counts) (40); and (b) the Inverse Simpson’s Index (ISI)) that measures both the number of different TCRs and their clonal expansion (Supplemental Table 2). The results showed that the TCRα and TCRβ repertoire richness of both CD4^+^ and CD8^+^ T cells varied greatly, ranging from 1 × 10^4^–10^6^ for both TCRα and TCRβ, with varying changes with age among the donors (Figure 2, A and C). For both CD4^+^ and CD8^+^ T cells, we found a significant reduction in richness with age for TCRβ but not TCRα and a significant reduction with age for both TCRα and TCRβ measured by ISI (indicating increased clonal expansion) using mixed linear effects (MLE) analysis (Figure 2, B and D). These findings suggested that repertoire changes with age affected both richness and clonal expansion, and that reduction in TCR repertoire richness was more rapid in CD8^+^ than in CD4^+^ T cells.

### Reductions in TCRα and TCRβ repertoires with age in naive and memory CD4^+^ and CD8^+^ T cells.

**Figure 3:**
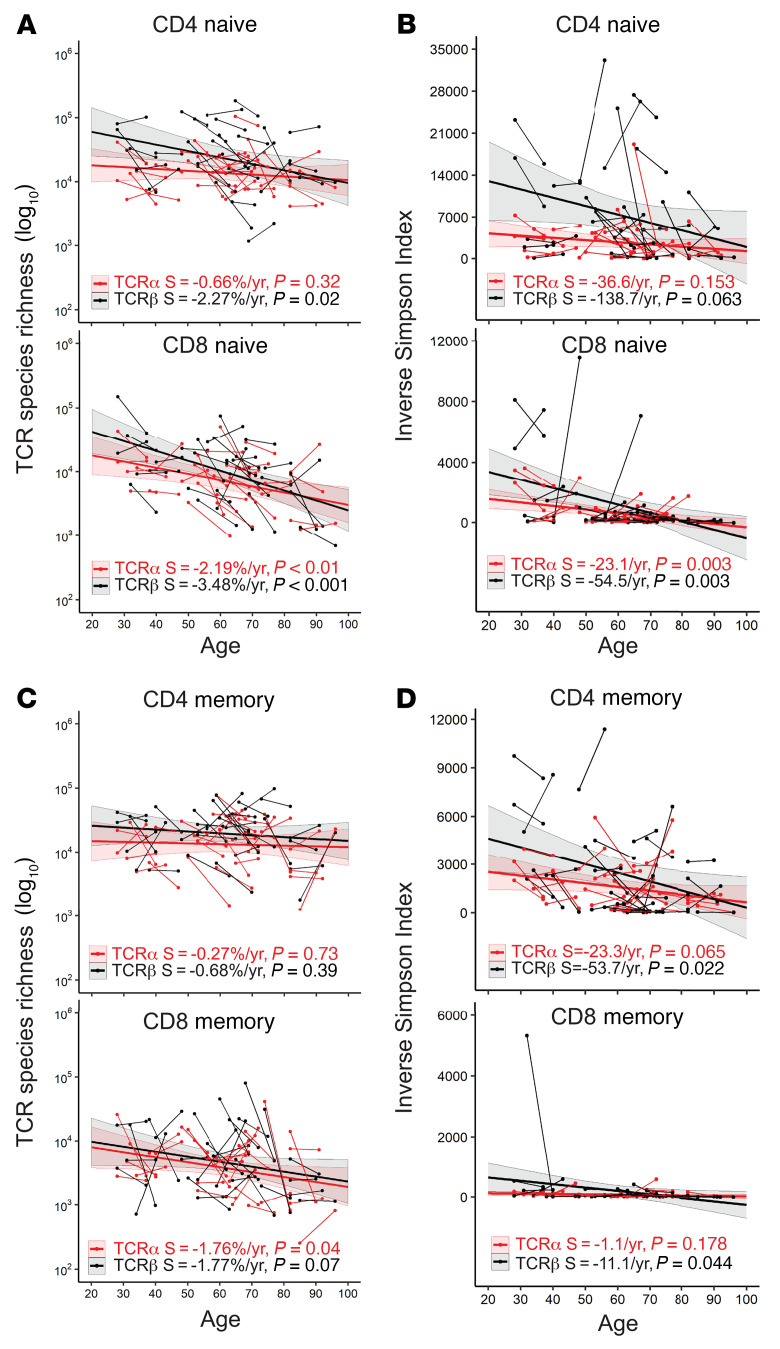
CD4^+^ and CD8^+^ T cell subset–specific reductions of αβ TCR repertoires with age. (**A**) Age-associated reduction in richness of TCRα and TCRβ repertoires of naive CD4^+^ and CD8^+^ T cells. TCRα and TCRβ sequences were calculated for each donor and projected for 1% of total circulating naive CD4^+^ or CD8^+^ T cells (in log_10_ -based values), for (A) and (C) in this figure. (**B**) Age-associated reduction in ISI for TCRα and TCRβ diversity of naive CD4^+^ and CD8^+^ T cells. (**C**) Age-associated reduction in richness of TCRα and TCRβ repertoires of memory CD4^+^ and CD8^+^ T cells. (**D**) Age-associated reduction in ISI of TCRα and TCRβ diversity of memory CD4^+^ and CD8^+^ T cells. Thin short lines link 2 donations from 1 participant. Thick lines are trends from MLE analysis. The colored shade around the trend line indicates the 95% confidence interval. S, slope of trend line.

The clonal distribution and expansion of naive T cells is an important determinant of T cell immunity (25). To determine whether the observed reductions with age in TCRα and TCRβ repertoires in CD4^+^ and CD8^+^ T cells occurred in naive or memory T cells, we measured TCRα and TCRβ repertoires of naive and memory CD4^+^ and CD8^+^ T cells isolated by cell sorting, with CD45RA^+^CD28^+^ cells sorted as naive and all other cells sorted as memory cells and determined changes in TCR repertoires with age. The richness of TCR repertoires was projected to 1% of the total actual naive and memory CD4^+^ and CD8^+^ T cells in the blood of donors, and TCR clonal expansion was calculated by ISI (Supplemental Table 3). We observed significant reductions with age in TCR repertoire richness, especially TCRβ richness, in CD4^+^ and CD8^+^ naive T cells, but not CD4^+^ and CD8^+^ memory T cells (Figure 3, A and C). Again, the reductions in TCR richness were more rapid in naive CD8^+^ T cells (TCRα = –2.19 %/year and TCRβ = –3.48%/year) than in naive CD4^+^ T cells (TCRα = –0.66%/year and TCRβ =–2.27%/year). Age also led to increased clonal expansion in the naive TCRβ repertoire of CD8^+^ but not CD4^+^ T cells (Figure 3, B and D). In contrast, reductions with age in TCRβ repertoire richness were not significant for CD4^+^ and CD8^+^ memory T cells (Figure 3C), but reduction of ISI with age was significant for TCRβ but not TCRα of memory CD4^+^ and CD8^+^ T cells (Figure 3D). Together, these findings demonstrated that age led to a more profound reduction in richness of TCRα and TCRβ repertoires in naive than in memory T cells. Age also resulted in a significant clonal expansion of TCRβ repertoires in both CD4^+^ and CD8^+^ naive and memory T cells.

Next, we analyzed TCR richness changes with age in naive and memory CD4^+^ and CD8^+^ T cells for each donor, comparing samples provided at different ages. To determine the true age-associated changes, we first measured TCR richness variation in samples collected at the same time but measured independently. The SDs of projected TCR richness of naive and memory CD4^+^ and CD8^+^ T cells were calculated using samples from 3 healthy adults (Supplemental Figure 2). We defined an age-associated change in TCR richness as greater than 1 SD in the estimated richness of each type of T cell subset. Using this criterion, we found the following changes in naive TCR repertoire richness among cell subsets. Reduced richness was observed in 59% of donors (average of TCRα and TCRβ for both naive and memory CD4^+^ and CD^+^ T cells); 11% had no obvious changes; and 30% had increased richness (Table 1). Further analysis showed that there was no statistical significance between the average age of donors in which their TCRα and TCRβ richness increased versus those in which their richness decreased (Supplemental Figure 3).

### Predicting paired αβ TCR repertoires and their age-associated changes.

**Figure 4:**
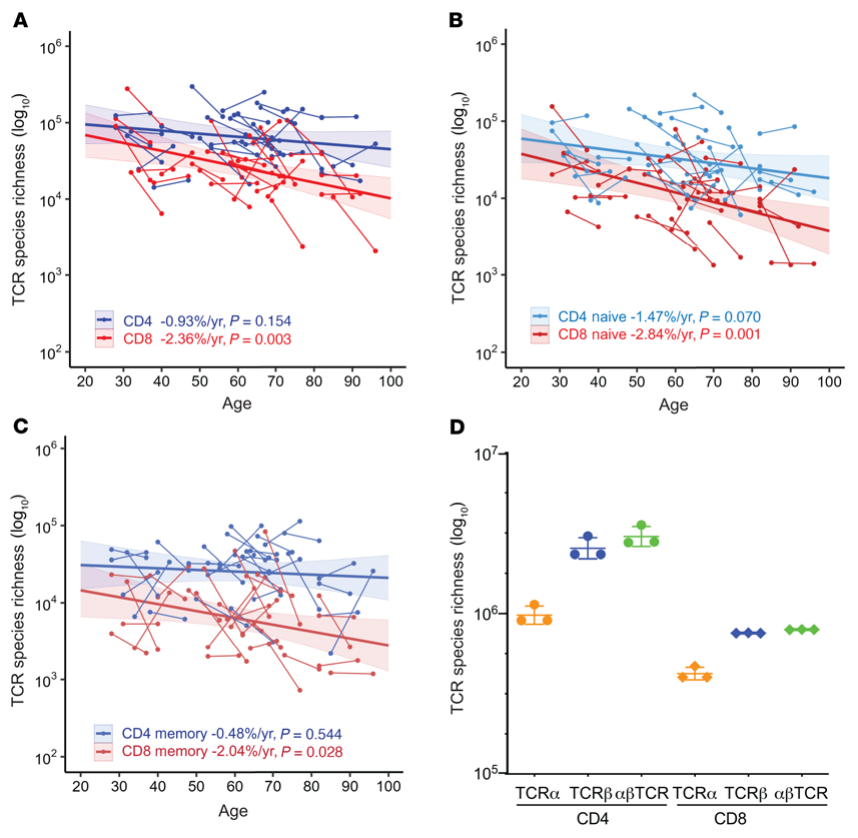
Age-associated decline of predicted αβTCR repertoires based on TCRα and TCRβ sequences. (**A**) Reduction with age of paired αβ T cell receptor (TCR) repertoire richness of CD4^+^ and CD8^+^ T cells. Paired αβ TCR repertoire richness of CD4^+^ and CD8^+^ T cells was estimated based on projected TCRα and TCRβ richness (1% of total circulating cells in blood) via linear regression of single-cell αβ TCR sequences (Supplemental Figure 4) (**A**–**C** of this figure). (**B**) Reduction with age in paired αβ TCR repertoire richness of naive CD4^+^ and CD8^+^ T cells. (**C**) Reduction with age of paired αβ TCR repertoire richness of memory CD4^+^ and CD8^+^ T cells. (**D**) Estimations of total αβ TCR clonotypes in CD4^+^ and CD8^+^ T cells. TCR data were combined across all 30 donors to predict richness. The estimated richness from the best 3 models are presented. Average values are 3.0 × 10^6^ for CD4^+^ T cells and 7.9 × 10^5^ for CD8^+^ T cells (projected to 1% of total cells in blood). For **A**–**C**, thin lines link the 2 donations from 1 participant. Thick lines are trends from MLE analysis. The colored shade around the trend line indicates the 95% confidence interval.

Studies have reported methods for pairing TCRα and TCRβ from bulk TCRα and TCRβ sequences using statistical modeling and frequencies (41, 42). We analyzed the relationship between separated TCRα and TCRβ sequences and their αβ-paired TCR using paired αβ TCR sequences (from 745,182 CD4^+^ and 158,305 CD8^+^ T cells) from single-cell RNA-seq studies and observed a linear relationship between the number of unique TCRα and TCRβ sequences and the number of paired αβ TCR clones (Supplemental Figure 4A). The numbers of TCRα and TCRβ and the numbers of their pairs reveal a mathematical principle that allows for direct estimation of αβ-paired TCR repertoires from individual TCRα and TCRβ sequences. Because some T cells have 2 functional TCRα sequences (43–45), we used the same data sets to calculate the average percentage of T cells with only single TCRα sequences and used this information to adjust the bulk TCRα sequences in calculations of paired αβ TCR richness (Supplemental Figure 4B). The TCR repertoire is larger for CD4^+^ T cells than for CD8^+^ T cells (19, 46), so we used separate equations to estimate the paired αβ TCR richness for CD4^+^ and CD8^+^ T cells (Supplemental Figure 4C). We found that projected paired αβ TCR richness was larger for CD4^+^ than CD8^+^ T cells, specifically an average 1.6-fold of the average TCRα and TCRβ richness for CD4^+^ cells and 1.5-fold of the average for CD8^+^ T cells (Figure 4 and Supplemental Table 2). Paired αβ TCR richness showed significant reductions with age for total CD8^+^ (–2.36%/year, *P =* 0.003), naive (–2.84%/year, *P =* 0.001), and memory (–2.04%/year, *P =* 0.028) CD8^+^ T cells, but not total, naive, or memory CD4^+^ T cells (Figure 4, A–C, and Supplemental Tables 2 and 3). To overcome the problem of a small number of T cells used for predicting the total TCR repertoire, we combined TCR sequences for CD4^+^ (1.26 × 10^8^) and CD8^+^ (6.07 × 10^7^) T cells for all 30 donors and projected αβ TCR richness for CD4^+^ and CD8^+^ T cells to 1% of the average of total cells in the blood for all donors. We found that the paired αβ TCR richness of 1% of average total blood was 3.0 × 10^6^ for CD4^+^ T cells and 7.9 × 10^5^ CD8^+^ T cells (Figure 4D). Thus, the αβ TCR repertoire richness in the total blood of an adult human was estimated, at the lower end, to be approximately 3.8 × 10^8^.

### Age-associated increased stability of TCRα and TCRβ repertoires.

**Figure 5:**
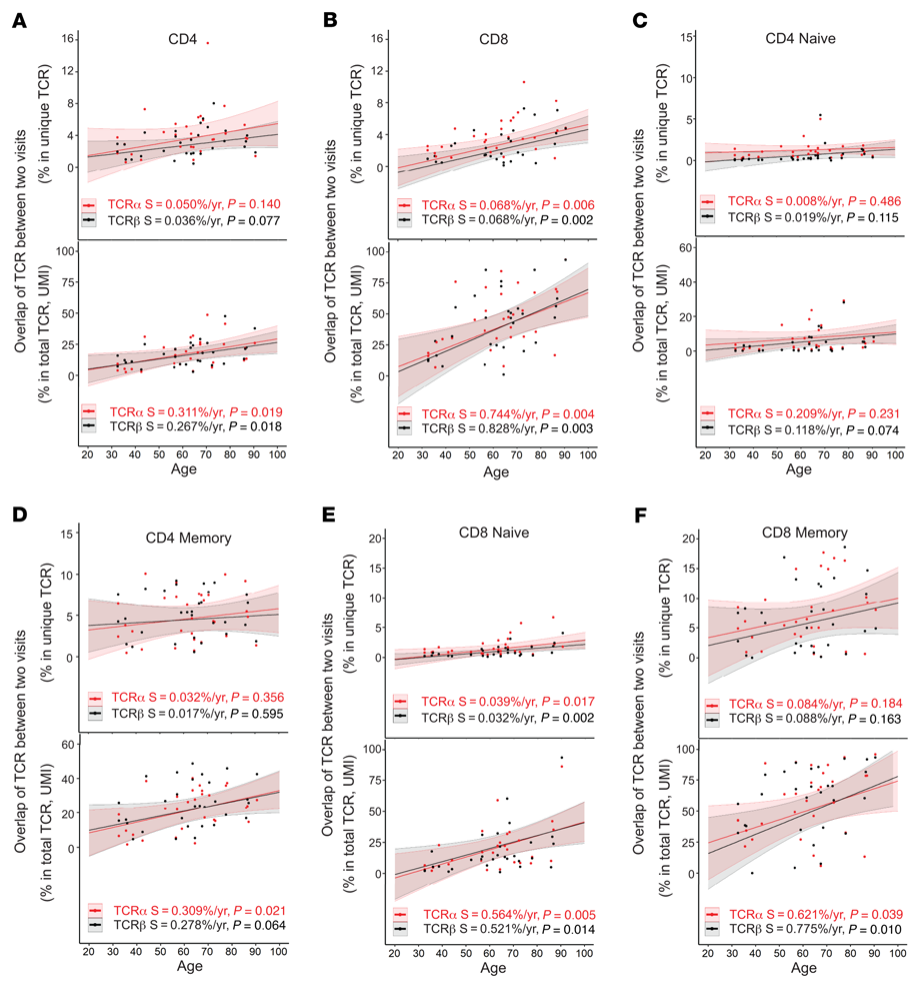
Increased stability of TCRα and TCRβ repertoires with age. (**A**) Increased overlapping TCRα and TCRβ sequences in CD4^+^ T cells with age. Percentages of overlapping TCR sequences in samples from 2 donations were calculated for unique (top) and total (bottom) TCR sequences and plotted by average donor age. For all graphs, thin lines link 2 donations from 1 participant, thick lines are trends calculated using linear regression analysis, and the colored shade around the trend line indicates the 95% confidence interval; S is slope with *P* values. (**B**) Increased overlap with age in TCRα and TCRβ sequences in CD8^+^ T cells. (**C**) Naive CD4^+^ T cells. (**D**) Memory CD4^+^ T cells. (**E**) Naive CD8^+^ T cells. (**F**) Memory CD8^+^ T cells.

We investigated how the content of TCRα and TCRβ sequences changes with age by analyzing the same TCR sequences that found in both first and second sample donations at both unique (reflecting changes in TCR richness level) and total (reflecting changes in T cell population level based on UMI counts) TCR sequences. We found that CD4^+^ and CD8^+^ T cells had a similarly low level of overlapping TCRs at young ages (<40 years old) with overlap increasing in older donors. This increase in TCR sequence overlap with age was more rapid for CD8^+^ than CD4^+^ T cells, particularly for total TCR sequences (Figure 5, A and B and Supplemental Table 4).

To determine if the age-associated increase in TCR overlap — defined as a TCRα or a TCRβ sequence observed in samples from both donations of a subject between (a) the same type of CD4^+^ and CD8^+^ T cells and their naive and memory subsets, and (b) between CD4^+^ and CD8^+^ T cells and their corresponding naive and memory subsets — was due to changes in naive or memory T cells, we compared naive and memory CD4^+^ and CD8^+^ T cells for overlap in TCR sequences using samples from participants’ 2 donations. In CD4^+^ T cells, memory cells had higher levels of overlapping sequences and a faster increase in overlap with age than naive cells for both unique (focusing on the changes at repertoire richness level) and total (focusing on the changes to repertoire richness at the cell level, which is influenced by clonal expansion) TCR sequences (Figure 5, C and D). Although the overlap was similar for naive and memory CD8^+^ and CD4^+^ T cells for younger donors (under 40 years), naive and memory CD8^+^ T cells exhibited a more rapid increase in TCR overlap with age, especially at the total TCR sequences (Figure 5, E and F). This effect was particularly profound in memory CD8^+^ T cells: TCR sequence retention over time was twice as high in older donors (8.8% and 9.1% for those over 70 years) as in young donors (4.1% and 4.4% for those under 40 years) at the unique TCRα and TCRβ levels. At the total TCR sequence level, the overlaps were substantially increased at both older (65% and 72% for TCRα and TCRβ, respectively) and younger ages (34% and 38% for TCRα and TCRβ) (Figure 5F). These findings demonstrated that (a) TCR repertoire was increasingly stable with increased age, (b) the TCR repertoire was more stable for CD8^+^ than CD4^+^ T cells with increased age, (c) the content of memory TCR repertoires showed greater increased retention with age than naive TCR repertoires, and (d) retained TCR sequences were more abundant than nonretained sequences with age.

### Age-reduced differences in TCRα and TCRβ repertoires in naive and memory CD4^+^ and CD8^+^ T cells.

**Figure 6:**
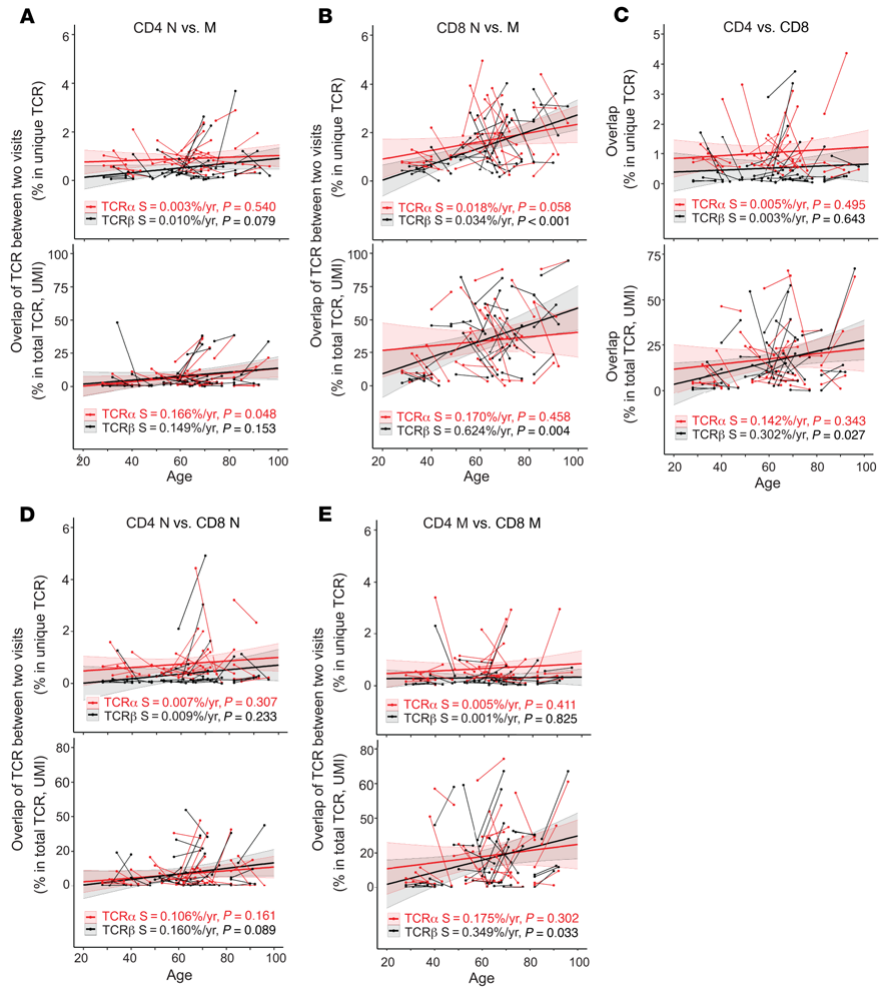
Age-reduced distinctness of TCRα and TCRβ sequences in CD4^+^ and CD8^+^ T cells, and naive and memory cells. (**A**) Changes with age in percent of overlapping TCRα and TCRβ sequences in naive (N) and memory (M) CD4^+^ T cells from a single sample, calculated at the unique (top) and total (bottom) TCR sequence level, plotted against donor age. (**B**) Increase in overlap of TCRα and TCRβ sequences between naive and memory CD8^+^ T cells with age. (**C**) Increase in overlap of TCRα and TCRβ sequences between CD4^+^ and CD8^+^ T cells with age. Data are from cells from a single sample for unique (left) and total (right) TCR sequences. (**D**) Overlap between naive CD4^+^ and CD8^+^ T cells. (**E**) Overlap between memory CD4^+^ and CD8^+^ T cells. Thin short lines link 2 donations from 1 individual. Thick lines are trend calculated by MLE analysis. The colored shade around the trend line indicates the 95% confidence interval. S, slope of trend lines, with *P* values.

Next, we compared TCRα and TCRβ sequences for cells from each participant for each sample donation to examine the degree of overlap between naive and memory TCR repertoire. We found low overlap of TCRα and TCRβ unique sequences between naive and memory T cells for CD4^+^ cells (the average of 2 donations was 0.9% for both TCRα and TCRβ) (Figure 6A and Supplemental Table 5) and CD8^+^ cells (1.3% and 1.4% for TCRα and TCRβ, respectively) (Figure 6B). But at the total TCRα and TCRβ sequence levels, there were more abundant overlapped TCR sequences in naive and in memory cells (7.2% and 8.0% of CD4^+^ and 34.7% and 34.3% of CD8^+^ total TCRα and TCRβ sequences, respectively) (Figure 6A–B).

Previous studies on the sharing of TCRα and TCRβ sequences between CD4^+^ and CD8^+^ T cells showed 9% sharing for TCRα and 1%–5% for TCRβ unique sequences (19, 46). Our study found little overlap in unique TCR sequences but an increased overlap with age in total TCR sequences in CD4^+^ and CD8^+^ T cells (Figure 6C and Supplemental Table 6). Analyzing the overlap between naive and memory CD4^+^ and CD8^+^ T cells, we found that the increased overlap in TCR sequences between CD4^+^ and CD8^+^ T cells was mainly in memory but not in naive T cells (Figure 6, D and E). These findings demonstrated that age resulted in a loss of distinctiveness in TCRα and TCRβ sequences between memory CD4^+^ and CD8^+^ T cells and suggested that overlapping TCRα and TCRβ sequences between memory CD4^+^ and CD8^+^ T cells were from selectively expanded TCRα and TCRβ clones. However, it requires further study to determine whether the increase in overlap with age is due to paired αβ TCRs or due to different αβ TCRs sharing identical TCRα or TCRβ sequences.

### Increased abundance of the public TCRα and TCRβ sequences with age.

**Figure 7:**
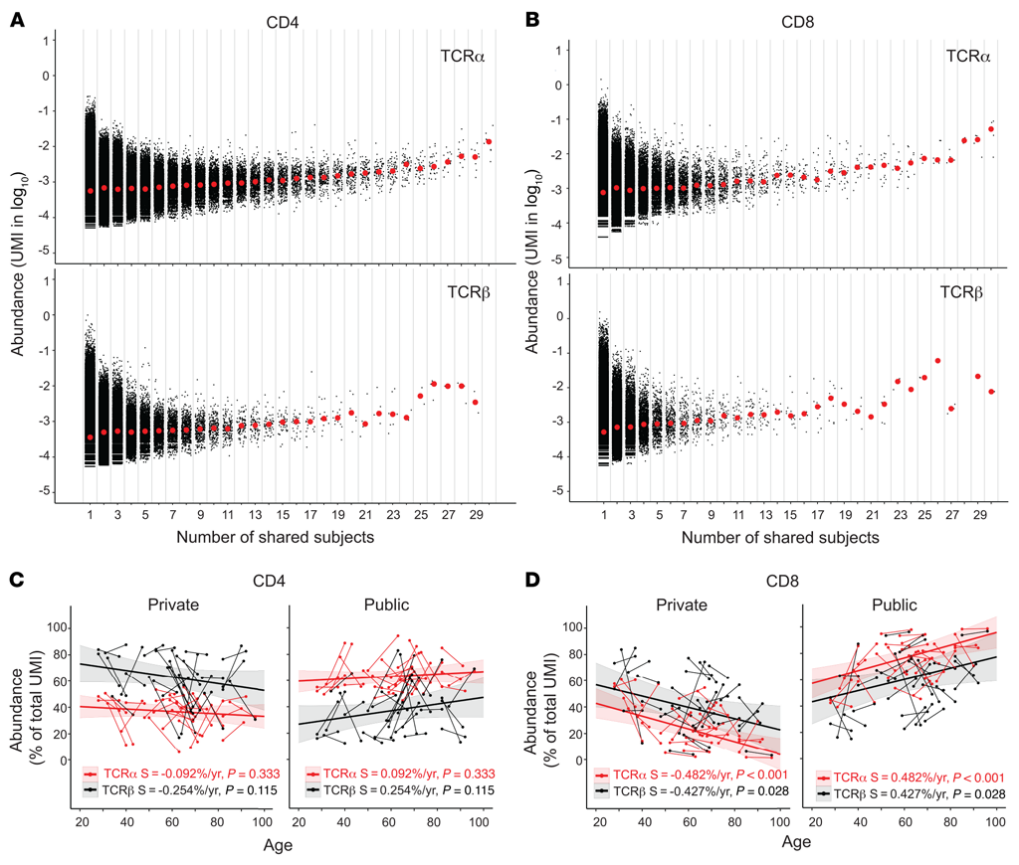
Abundance of private and public TCRα and TCRβ sequences in CD4^+^ and CD8^+^ T cells and changes with age. (**A**) Abundance of private (found in 1 donor only) and public (found in 2–30 donors) TCRα (top) and TCRβ (bottom) sequences of CD4^+^ T cells. Black dots are number of UMIs for 1 TCRα or TCRβ sequence. Red dots are median values for TCRs that share the same number of donors (in log_10_). (**B**) Abundance of private and public TCRα (top) and TCRβ (bottom) sequences of CD8^+^ T cells. (**C**) Alteration of the abundance of private and public TCRα and TCRβ sequences of CD4^+^ T cells with age. Private and public TCRα and TCRβ sequences of CD4^+^ T cells for each donor. Each sample is presented as percentage of total TCRα and TCRβ sequences (UMI counts). Thin short lines link 2 donations from 1 individual. Thick trend lines were calculated by MLE analysis. The colored shade around the trend line indicates the 95% confidence interval. Slope (S) and *P* values are provided. (**D**) Alteration of the abundance of private and public TCRα and TCRβ sequences of CD8^+^ T cells with age.

Retention of TCR sequences within an individual over time suggests that these TCR clones are useful to that individual, whereas presence of a common TCR sequence among different individuals implies a common pathogen exposure among the individuals. To determine the degree of TCR sequences shared among different individuals, we analyzed the sharing of TCR sequences among the 30 healthy adult donors. We defined a TCR sequence as a unique combination of V-CDR3-(amino acid sequence)-J. Any TCR sequence found in only 1 donor was “private” and sequences found in more than 1 donor were “public.” The degree of the publicity of a TCR sequence was determined by the number of individuals who shared it. The publicity of TCRα and TCRβ sequences was associated with their abundance in both CD4^+^ and CD8^+^ T cells (Figure 7, A and B). Our findings showed that the abundance of the public TCRs increased with age in both CD4^+^ and CD8^+^ T cells and that the increase was significant in CD8^+^ but not in CD4^+^ T cells (Figure 7, C and D). This result suggested a more profound expansion of public TCR sequences with age in CD8^+^ than in CD4^+^ T cells. The antigenic feature of these public TCR sequences enriched in old adults requires further study.

## Discussion

Our study used longitudinal samples of total CD4^+^ and CD8^+^ T cells and their subsets and predicted repertoires based on the actual blood T cell number of each donor. We demonstrated that TCR repertoire reduction with age is specific to different T cell subsets and occurs at an individualized rate. With age, naive T cells show reduced TCR repertoire richness, while memory T cells show increased clonal expansion. Our study documents age-associated changes in the αβ TCR repertoires of naive and memory CD4^+^ and CD8^+^ T cells of healthy adults over a span of 70 years and provides evidence of a reduced TCR repertoire in older adults.

Richness and clonal distribution/expansion are 2 key features of TCR repertoires. Although previous cross-sectional analyses suggest a reduction in TCR repertoire richness with age (21, 23), it was unknown if richness and clonal distribution/expansion changed in parallel to or independently of age. Our longitudinal study observed an age-associated reduction in TCRα and TCRβ richness and ISI in both CD4^+^ and CD8^+^ T cells and in both their naive and memory cell subsets. Although expanded TCR clones were recently observed in human naive T cells (25), our findings showed that clonal expansion of certain TCRs increases with age (seen as a reduction of ISI with age) in both CD4^+^ and CD8^+^ naive T cells, providing strong evidence that age alters the homeostatic maintenance of naive T cells. In contrast, CD4^+^ memory T cells did not show age-associated reduction in TCR richness, but did show significant reduction in ISI (particularly in TCRβ), indicating different aspects of TCR repertoire change with age in naive and memory T cells. Advancing age has the most dramatic effect on naive CD8^+^ T cells: age reduces cell number (10, 47) and TCRα and TCRβ repertoire richness. Here, we showed that age also altered their homeostasis with expansion of selected TCRs in naive CD8^+^ T cells. Evidently, TCR repertoire age-related changes are influenced by multiple factors such as history of infections, genetic elements including HLA haplotypes — 50% of the study participants here are HLA-A2^+^ — and other differences among the participants. Chronic CMV infections drive oligoclonal expansions of CD8^+^ T cells in old age (48, 49) but we did not have the power to address whether this virus may have been a confounder, potentially preferentially affecting clonal expansions in the CD8^+^ T cell memory compartment of older participants, as only around 50% of our participants indicated their CMV status. We also noticed dissociated age changes of TCRα and TCRβ richness between naive and memory CD4^+^ and CD8^+^ T cells in individual participants. Whether this is a small sampling error or reflects a yet-to-be determined type of age-related change remains to be elucidated. Clearly, more studies will be needed to determine the contribution of the effect of chronic infections on the rates of TCRα and TCRβ repertoire richness and diversity changes using condition matched and naive participants.

Previous phenotype-based flow cytometry analyses of T cell subsets provided insights into changes at the cell population level (10, 47) but lack information about TCR content changes with age. Our longitudinal αβ TCR sequence analysis by defined T cell subsets shows the dynamics of TCRα and TCRβ sequence changes with age in adults and reveals some intriguing findings. First, with age, TCR sequences were increasingly retained (meaning the same TCRs were found in both samples from an individual). This was found for both CD4^+^ and CD8^+^ T cells with a more prominent degree of retention in CD8^+^ cells than CD4^+^ cells. Second, the retention of TCR sequences was more obvious in memory than in naive T cells, with the highest retention of TCR sequences in memory CD8^+^ T cells. These findings demonstrate that the content of TCR repertoires is increasingly stabilized as repertoire size reduces with advancing age, particularly in memory CD8^+^ T cells. Although the precise loss of the kind of TCRs was unknown, this reduction provides an explanation of reduced ability against novel antigens in older adults.

Another striking difference between CD4^+^ and CD8^+^ T cells was a greater increase with age in the overlap of TCR sequences with total sequences between naive and memory cells in CD8^+^ than in CD4^+^ cells. In donors over 70 years old, the sharing of identical TCR sequences between naive and memory cells was 4–5 times greater in CD8^+^ T cells (42% and 45% for total TCRα and TCRβ, respectively) than in CD4^+^ cells (8% and 10%). This finding implies that either the phenotypic definition of naive T cells is more stable in CD4^+^ than in CD8^+^ T cells with age, or that the TCRs of naive and memory cells undergo more parallel selection in CD8^+^ than in CD4^+^ T cells in older individuals. More studies are needed to understand the mechanisms and implications underlying these changes.

In addition, we observed a reduction with age in the distinctiveness of TCR sequences between CD4^+^ and CD8^+^ T cells. Intriguingly, although an increasing overlap was observed in TCR sequences in both naive and memory cells, the overlap between naive CD4^+^ and CD8^+^ T cells (8.1% and 7.3% of total TCRα and TCRβ, respectively) was less than twice the overlap between memory CD4^+^ and CD8^+^ T cells (18% and 16%). Although it is highly unlikely that the sorted naive and memory CD4^+^ and CD8^+^ T cells in our experiments were contaminated with an increasing number of CD4^+^/CD8^+^ double-positive T cells with age, some dysregulation of CD4 and CD8 expression may have occurred in the memory T cells of our study participants. It is currently unclear if this increased overlap of TCRs exhibits the self-reactivity (50). The underlying mechanisms causing the increased sharing of TCR sequences between CD4^+^ and CD8^+^ T cells with age and how such changes effect T cell function in older adults requires further study.

Despite recent progress in deep sequencing, accurately estimating the αβ TCR repertoire in humans using a small fraction of cells (~1 × 10^6^) is challenging, given the immense number of total T cells in the human body. Current single-cell methods for paired αβ sequencing of TCRs have a capacity of less than 1 × 10^4^ cells per sample. Measurements of TCRα and TCRβ sequences separately have a 100-fold larger capacity of 1 × 10^6^ per sample, but lack information about αβ pairing on TCRs. We developed equations based on single cell αβ paired TCR data to calculate paired αβ TCR richness using bulk TCRα and TCRβ sequences. Our lower estimate of the αβ TCR repertoire richness of T cells in a healthy adult using combined TCR sequences from all study donors was approximately 3.8 × 10^8^ (3.0 × 10^8^ for CD4^+^ and 7.9 × 10^7^ for CD8^+^ T cells). Considering that the number of cells used for estimation was still 4 orders of magnitude lower than the total number of T cells in an adult human, these numbers likely underestimate the actual αβ TCR repertoire richness.

In conclusion, we show that T cell subsets display distinct age-related TCR repertoire changes and that the CD8^+^ TCR repertoire reduces with age more profoundly than the CD4^+^ TCR repertoire by an RNA-based UMI-corrected method. It will be worth comparing the findings with the DNA-based TCR-sequencing method, which is not influenced by the potential variance in TCR copy number (51). Consequently, the CD8^+^ TCR repertoire is increasingly smaller and more stable in older adults. Thus, understanding the kinds of TCRs that are lost during aging could reveal specific weaknesses in the T cell immunity of aging individuals and open new avenues for developing tailored immunotherapy to specific immunity defects in the population of older adults.

## Methods

### Selection of study participants.

We selected 30 healthy participants (15 male and 15 female participants, ages ranging from 28 to 85 years old) from the Baltimore Longitudinal Study of Aging (BLSA), an ongoing prospective observational study of normative aging in community-dwelling volunteers. Demographic characterization is in Supplemental Table 1. During each visit, body weight, height, and blood cell counts using standard CBCs were measured; and PBMCs were isolated and cryopreserved in a liquid nitrogen freezer. Each donor had 2 visits separated by an average of 9 years (range 7–13 years). The proportions of T cells and PBMC subsets were determined by flow cytometric analysis (see gating strategy in Figure 1) and used with CBCs to calculate the number of T cells in a microliter of blood. The total blood volume was calculated based on weight and height using Nadler’s formula (39). The numbers of T cells and subsets in the total blood of each donor were calculated based on blood volume.

### Isolation of naive and memory CD4^+^ and CD8^+^ T cells.

Frozen PBMCs from both of a donor’s visits were thawed at the same time and resuspended in RPMI1640 with 10% fetal bovine serum containing L-glutamine (0.3 mg/ml), penicillin (50 units/ml), and streptomycin (50 μg/ml) (Thermo Fisher) and incubated at 37°C overnight before cell sorting. The following day, PBMCs were collected, counted, and stained with antibodies against CD3, CD4, CD8, CD45RA, and CD28 (Biolegend) (catalog numbers available in Supplemental Table 7). Naive CD4^+^ and CD8^+^ T cells were defined by CD45RA^+^CD28^+^ and the remaining cells were sorted as memory T cells (Figure 1). Subsets of memory T cells were further analyzed by expression of CD45RA and CD28 and divided into central (Tcm), effector (Tem) and effector memory expressing CD45RA (Temra) subsets. The purity of sorted cells was over 95%, and cells were counted and lysed immediately for RNA isolation.

### Library construction and sequencing strategy.

The αβ TCR cDNA library construction was described previously (37). Total RNA was isolated from sorted CD4^+^ and CD8^+^ T cell subsets (naive and memory) using a Qiagen RNeasy Micro kit. Up to 500 ng total RNA was used for cDNA synthesis using specific primers to TCRα and TCRβ constant regions (ac1R and bc1R), SMARTScribe reverse transcriptase (Takara Bio), and SmartN oligos for template switching at the 5′ end to incorporate a UMI and M1SS sequence for PCR (Supplemental Table 2). The cDNA products were treated with uracil-DNA glycosylase (New England Biolabs) at 37^o^C for 30 minutes to remove SmartN oligos (Supplemental Table 8). We applied 3 rounds of PCR using high fidelity Platinum *Taq* DNA polymerase (Thermo Fisher) to prepare libraries for sequencing. The amount of purified DNA was measured using an Agilent BioAnalyser or Qubit, and samples with distinct barcodes were combined for sequencing. The amount of DNA used for sequencing was based on the number of cells in each sample, and different samples were combined for the total needed sequence reads close to the size of sequencing capacity. Sequencing was performed with 50 pM of combined DNA were used on an Illumina HiSeq 2500 system. A modified paired-end sequencing protocol was used: TCR-specific sequencing primers TRA and TRB (Supplemental Table 8) were used for first round sequencing of 150 bps. Illumina RD2 primer was used for second round, sequencing of 50 bps, covering the sample barcode and UMI.

### Identification of TCRα and TCRβ sequences.

Samples were separated after identifying sample barcodes and UMIs from raw sequence reads from the Illumina Sequencer using a custom Demultiplexor Python script. The FASTQ files generated from this script were reformatted to meet standard MiGEC FASTQ conventions and tagged with the PCR amplification time through custom Python scripts. Sequences from the same donor were combined and processed through a custom Python script to account for PCR amplification times (for contamination analysis) and to separate conflicting TCR sequences under identical UMIs. To identify TCRα and TCRβ sequences, we used MiGEC (v. 1.2.7) to determine V/J genes and CDR3 amino acid and nucleotide sequences (36). Consensus sequences were assembled using a minimum of 3 reads per UMI (*-m*) and a UMI quality score filter of 10 (-*q*) using the *Assemble* function. Consensus sequences were mapped by specifying “TRA,TRB” for the desired genes argument (*-R*) and “HomoSapiens” for the species argument (*-S*) using the *CdrBlast* function. Identified TCRα and TCRβ sequences were further cleaned by removal of CDR3s with stop codons and summarized for each donor. Final functional TCR sequences were required to have at least 3 sequence reads. A unique TCR sequence was defined as a unique combination of V, J, and CDR3 amino acid sequences.

### Removal of potential contaminating TCR sequences.

The chance of the same UMI and TCR sequence combinations from different donors was estimated to be less than 1 in 1 × 10^10^. (The diversities of UMI and TCR in a typical sample were approximately 1 × 10^6^ and 1 × 10^4^, respectively). When the same UMI (12 bp) and TCRα or TCRβ sequence was found in different samples, we considered the TCRα or TCRβ sequence to be contaminated. Any TCR sequences with identical UMIs between 2 or more samples or donors were assessed based on when they were PCR amplified; TCRs with the earliest PCR amplification time were retained and the remaining TCRs were removed. If multiple samples shared the same early PCR time for a given TCR, the TCRs were removed from subsequent analysis. Contamination analysis was performed on the MiGEC BLAST-converted files using a series of custom Python scripts.

### Measurement of the TCR repertoire of samples collected at the same time.

Apheresis blood from 3 healthy adults was collected under an IRB-approved protocol. Naive and memory CD4^+^ and CD8^+^ T cells were isolated by cell sorting using the same phenotypic markers as for frozen PBMCs described above. Sorted naive and memory CD4^+^ and CD8^+^ T cells were aliquoted to the same number of cells (from 0.2-1 million) per vial with 3 to 4 vials per person. TCRα and TCRβ sequences were determined for each sample. DivE (40) was used to calculate the richness of TCRα and TCRβ sequences of each sample, and SDs for each type of cell and average percentages were calculated.

### Species richness estimated by DivE method.

The DivE R package (v.1.0) was used to analyze species richness (40). Fifty models were used to concurrently estimate the number of unique TCR sequences for each donor. Two subsamples (*divsubsamples*) were created, 1 spanned to a normalized-UMI count and the other spanned to half of the normalized count. Each subsample used the following parameters: 1000 subsamples (*NResamples*), and a rarefaction length of 2000 (*nrf*). Both subsamples were passed to the *DiveMaster* wrapper function with the following parameters: 2 fit-loops (*fitLoop*), and 100 as an optimization parameter (*numit*). This wrapper function requires a total population number (T cell population) to estimate the species richness; we calculated the T cell population counts based on staining results and physical characteristics of the donor (Supplemental Table 1). We then adjusted these population counts by a factor of 1/100 and passed this adjusted number into the *tot.pop* argument in the wrapper function, due to limited computational resources. All models with a score above 250 were filtered out, and the 3–5 models with the lowest scores were used for calculation of the average. The geometric mean of top diversity estimates was presented as estimated species richness.

### ISI.

The ISI was used to calculate the species abundance of the TCR repertoire (52). The index was calculated as (Equation 1):
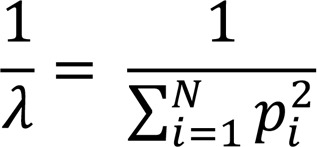


where *i* represents each TCR sequence, *N* represents the number of TCR sequences for a given sample, *p_i_* represents the UMI percentage occupancy for a TCR (UMI count of TCR divided by total UMI counts), and λ represents Simpson’s index.

### Overlap analysis.

Overlap analysis was defined using a TCR sequence as a unique combination of V-CDR3 AA-CDR3 NT (nucleotide sequence)-J. The unique TCR repertoire percentage overlap (focusing on the changes at repertoire richness level) between samples A and B was calculated using the following equation (Equation 2):
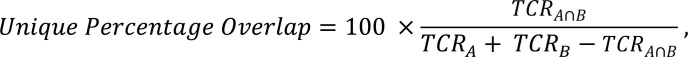


where *TCR_A∩B_* is the number of TCRs found in both samples A and in B, *TCR_A_* is the total TCR count for sample A, and TCR*_B_* is the total TCR count for sample B. The total TCR repertoire percentage overlap (focusing on the changes in repertoire at the cell level, which is influenced by clonal expansion) between samples A and B was calculated using the following equation (Equation 3):
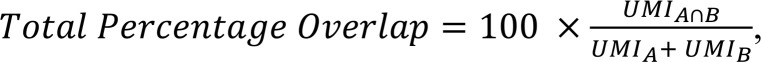


where *UMI_A∩B_* is the UMI count for TCRs found in both samples A and in B, *UMI_A_* is the total UMI count for sample A, and *UMI_B_* is the total UMI count for sample B.

### Public TCR analysis.

The UMI counts for all CD4^+^ or CD8^+^ αβ TCRs for a given donor were cumulatively added together and the UMI percentage for each TCR was calculated. The median UMI percentage was calculated for each unique TCR across all donors and adjusted via log_10_ reduction. Regardless of whether a TCR was from an individual’s first or second donation, it was considered private only if it was found in only a single donor’s repertoire and considered public otherwise.

### Estimating paired αβ TCR richness based on unpaired TCRα and TCRβ richness.

Because TCRα and TCRβ sequences were determined separately, the actual pairing of α and β in TCRs of the samples was unknown. Establishing a general correlation between separated TCRα and TCRβ sequences and paired TCR sequences allowed us to estimate the paired TCR richness from the richness in the separated TCRα and TCRβ sequences. To achieve this, we collected human single-cell paired αβ TCR data from 9 publicly available data sets (GSE107646 (53), GSE108989 (54), GSE114724 (55), GSE137275 (56), and GSE100378 (57), PRJNA593622 (58), PRJCA001702 (59), Github Repository at https://github.com/JasonACarter/CD4_CD8-Manuscript (46), and 10X Genomics at https://support.10xgenomics.com/single-cell-gene-expression/datasets). Paired TCRs from a total of from 745,182CD4^+^ T cells and 158,305 CD8^+^ T cells were used for analysis. Samples containing fewer than 2,000 cells were merged to ensure that single-cell sample sizes more closely resembled our TCRα and TCRβ samples. In addition, CD8^+^ paired TCRs extracted from 15 total T cell samples (58) via a random forest model trained to discriminate between CD8^+^ and CD4^+^ TCRα and TCRβ (19) were included in our single-cell αβ TCR data set. Sample merging and extraction resulted in 25 samples for CD4^+^ and 26 samples for CD8^+^ T cells.

To establish the above correlation in this data set, the number of unique paired sequences in each single-cell sample was plotted against the sum of its unpaired TCRα and TCRβ sequences. Two adjustments were made prior to linear regression. First, the larger of unique TCRα and TCRβ sequences were identified for each sample and subtracted from the sample’s unique paired sequences count. Second, the linear fit was made with a fixed y-intercept at Y=0. These 2 adjustments guarantee that paired αβ TCR projections are not smaller than the number of either of the unpaired sequences. This linear relationship was calculated as follows: αβ *TCR* − *MAX*(*TCRα, TCRβ*) = *M* × (*TCRα* + *TCRβ*) + 0, where αβ TCR indicates all samples’ paired richness, and TCRα and TCRβ indicate their unpaired richness. *M* is calculated as the best linear fit to these data. Linear regression was carried out via Scientific Computing with Python (SciPy) on the CD4^+^ and CD8^+^ T cell samples separately. Fitting results were recorded and presented (Supplemental Figure 4A). This plot demonstrates the ability of unpaired TCRα and TCRβ richness to predict αβ TCR richness using the equation above. Note that the TCRα richness values of the above equation must be preceded with a corrective coefficient, as below.

A single T cell sometimes expresses 2 distinct TCRαs (43–45). While single-cell sequencing can resolve the primary and secondary TCRαs, bulk-separated TCRα sequencing is unable to distinguish them. To avoid overestimating αβ TCR richness from bulk unpaired TCRα sequences containing secondary TCRαs, we determined the frequency of T cells with 2 distinct TCRα sequences in the data set for CD4^+^ and CD8^+^ T cells (Supplemental Figure 4B). On average, single TCRα was found in 87.0% of CD4^+^ cells and 84.6% of CD8^+^ cells. This information was used to adjust the nonsingle-cell sequenced TCRα richness in αβ TCR estimation equations for CD4 and CD8 listed below (Supplemental Figure 4C). *CD*4: *αβ* *TCR* = 0.138 × (0.870 × *TCRα* + *TCRβ*) + *Max* (0.870 × *TCRα*, *TCRβ*); *CD*8: *αβ* *TCR* = 0.035 × (0.846 × *TCRα* + *TCRβ*) + *Max* (0.846 × *TCRα*, *TCRβ*)

### Data availability.

Sequence data were deposited at SRA (PRJNA602091) and custom scripts for data analysis were deposited at Github (https://github.com/Weng-lab-NIH/TCR_Longitudinal_Aging; commit ID f365444ad6a19d737ef4c133218e2573022a8cae).

### Statistics.

All regression and statistical analyses used R (v.3.6.1). Longitudinal data were analyzed via a MLE model, with the measured value as a function of age and sex with the donor as the random intercept; statistics associated with the MLE model were calculated using the *nlme* package (v.3.1-140). For the TCR species richness and total projected cell counts data, we first log_10_ transformed the data and then applied an MLE model to the transformed response. Slope values for these data were recalculated based on the following equation for better interpretation (60, 61): *S_new_ =* (10*S_old_* – 1) × 100, where *S_old_* is the slope of the model based on log_10_ values and *S_new_* represents the slope of the model expressed as percent change of the original response variable per year. Linear regression data were analyzed via a simple linear regression model, with the measured value as a function of age and sex. *P* value statistics were calculated using the *stats* (v.3.6.1) package. *P* values of less than 0.05 were considered significant.

### Study approval.

BLSA and the human studies were approved by the Intramural Research Program of the US National Institute on Aging and the Institutional Review Board of the National Institutes of Health. All participants provided written, informed consent at every visit prior to the blood draw.

## Author contributions

XS and AA carried out most experiments; TN, AK, JC, CL, JS, and YZ did all computational and statistical analysis of the data; TH, WW, WWW, JNP, KGB and RFS helped with experiments and deep sequencing; CWC, LZ, and LF helped with donor selection and sample collections; and XS, AA, TN and NPW designed experiments and wrote the manuscript.

## Supplementary Material

Supplemental data

Supplemental tables 1-8

## Figures and Tables

**Table 1:**
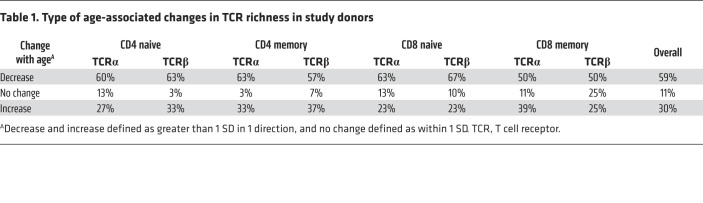
Type of age-associated changes in TCR richness in study donors

## Version 1 06/16/2022

In-Press Preview

## Version 2 09/01/2022

Electronic publication
