# Supplementary material for: Longitudinal analysis reveals age-related changes in the T cell receptor repertoire of human T cell subsets
Source: J Clin Invest. 2022 Sep 1;132(17):e158122. doi: 10.1172/JCI158122 (PMC9433102; doi:10.1172/JCI158122)
Supplement: Supplemental data [file jci-132-158122-s111.pdf]

Age-related changes in the TCR repertoire of human naïve and memory $CD4^+$ and $CD8^+$ T cells by longitudinal analysis

Xiaoping Sun[1*], Thomas Nguyen[1*], Achouak Achour[1*], Annette Ko[1], Jeffrey Cifello[1], Chen Ling[1], Jay Sharma[1], Toyoko Hiroi[1], Yongqing Zhang[2], Chee W. Chia[3], William Wood, III[2], Wells W. Wu[4], Linda Zukley[5], Je-Nie Phue[4], Kevin G. Becker[2], Rong-Fong Shen[4], Luigi Ferrucci[5], and Nan-ping Weng[1**]

[1] Laboratory of Molecular Biology and Immunology, National Institute on Aging, NIH, Baltimore, MD, USA

[2] Gene expression and Genomics Unit, Laboratory of Genetics and Genomics, National Institute on Aging, NIH, Baltimore, MD, USA

[3] Laboratory of Clinical Investigation, National Institute on Aging, NIH, Baltimore, MD, USA

[4] Facility for Biotechnology Resources, CBER, Food and Drug Administration, Silver Spring, MD, USA

[5] Translational Gerontology Branch, National Institute on Aging, NIH, Baltimore, MD, USA

Supplemental Figure S1: Changes of T cell counts in blood and memory T cell subsets with age.

Supplemental Figure S2: Variations of TCRα and TCRβ richness of naïve and memory $CD4^+$ and $CD8^+$ T cells isolated at the same time.

Supplemental Figure S3: CDR3 lengths of TCRα and TCRβ in total, naïve and memory $CD4^+$ and $CD8^+$ T cells in study subject.

Supplemental Figure S4: Development of equations to predict paired αβ TCR richness based on the separated TCRα and TCRβ sequences.

Supplemental Table S1: Demographics of study subjects and their blood T cell subset counts

Supplemental Table S2: Summary of αβ TCR repertoire of $CD4^+$ and $CD8^+$ T cells

Supplemental Table S3: Summary of αβ TCR repertoire of naïve and memory $CD4^+$ and $CD8^+$ T cells

Supplemental Table S4: Overlap of TCR sequences between two visits

Supplemental Table S5: Overlap Percentages of TCR sequences between naïve and memory $CD4^+$ and $CD8^+$ T cells

Supplemental Table S6: Overlap of TCR sequences between $CD4^+$ and $CD8^+$ T cells

Supplemental Table S7: Oligos used in TCR library construction and sequencing

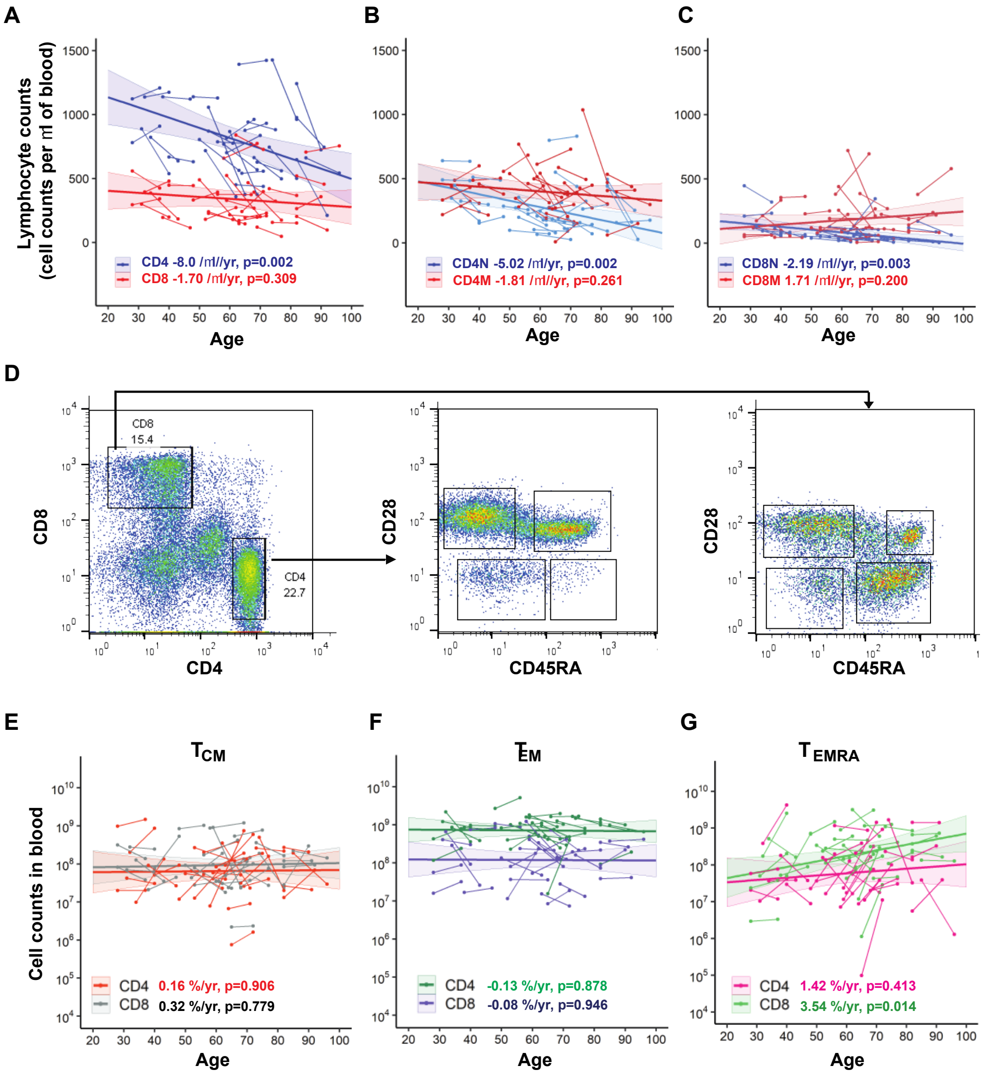

Supplemental Figure 1: Changes of T cell counts in blood and memory T cell subsets with age. A. Changes in $CD4^+$ and $CD8^+$ T cell counts in blood with age. Cell counts were calculated based on 1) lymphocyte counts per microliter of blood and 2) percentage of $CD4^+$ and $CD8^+$ T cells and naïve ($CD45RA^+CD28^+$) and memory cells in lymphocytes, calculated from flow cytometry. D. Gating of $CD4^+$ and $CD8^+$ T memory T cell subsets: T_{CM}($CD45RA^-CD28^+$), T_{EM}($CD45RA^-CD28^-$), and T_{EMRA}($CD45RA^+CD28^-$). E-G, Numbers of $CD4^+$ and $CD8^+$ T_{CM}, T_{EM}, and T_{EMRA} cells in blood at each visit and their change with age.

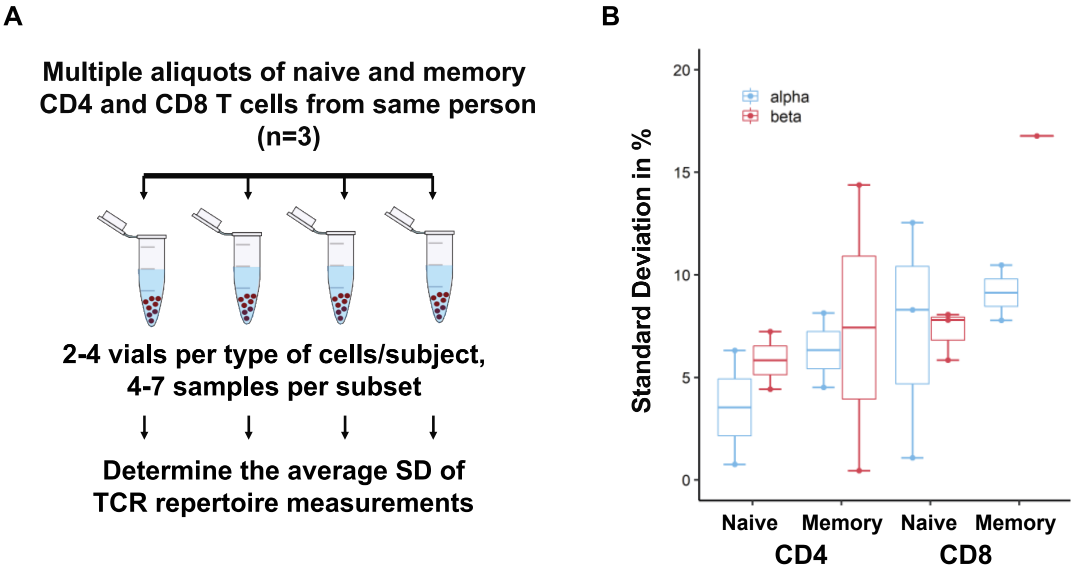

Supplemental Figure 2: Variations of TCRα and TCRβ richness of naïve and memory $CD4^+$ and $CD8^+$ T cells isolated at the same time. A. Experimental plan. Naïve and memory $CD4^+$ and $CD8^+$ T cells were isolated from blood (three healthy adults) by cell sort and equal number of sorted cells (0.5-1 x 10^6) were aliquot in 3-4 tubes. TCRα and TCRβ sequences in each tube were independently measured and then predicted to 10^7 by DivE method. B. Standard deviations of projected richness of TCRα and TCRβ are presented as percentages.

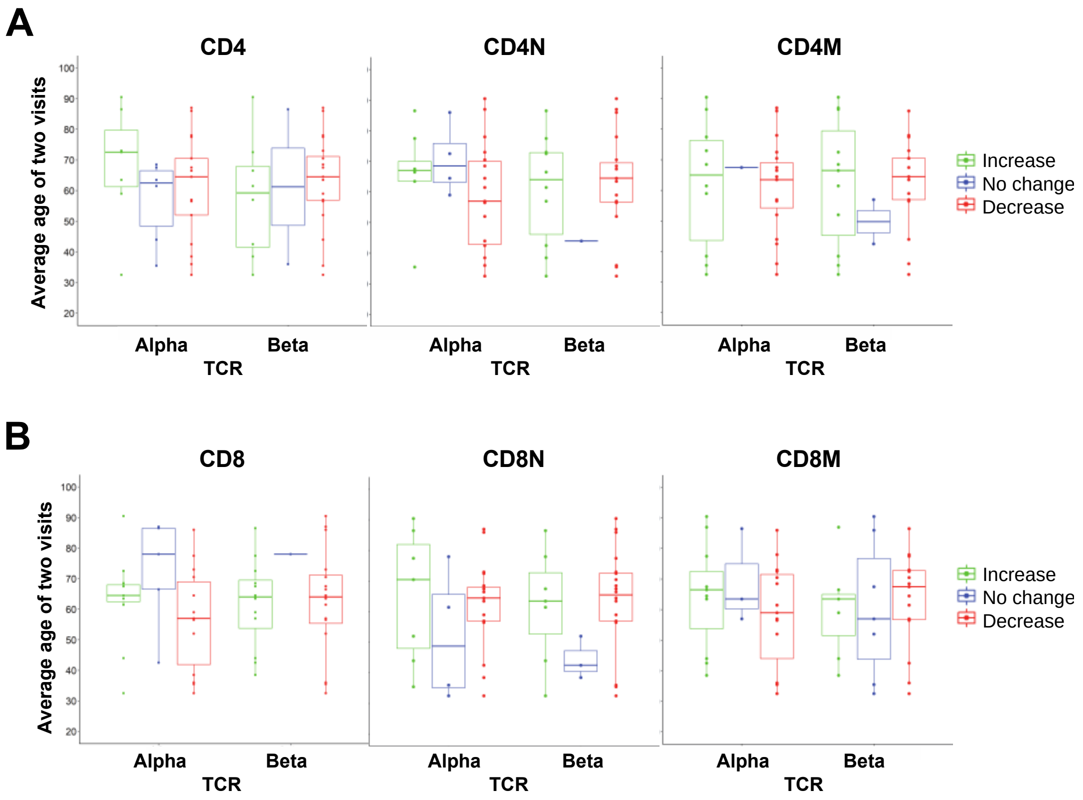

Supplemental Figure 3: Average age of three groups of TCRα and TCRβ richness changes in in total, naïve and memory $CD4^+$ and $CD8^+$ T cells. A. Box-Whisker plot of average age of three groups (decrease, no change, and increase) of richness changes with age in total, naïve (N), and memory (M) $CD4^+$ T cells.. B. Box-Whisker plot of average age of three groups of richness changes with age in total, naïve (N), and memory (M) $CD8^+$ T cells.

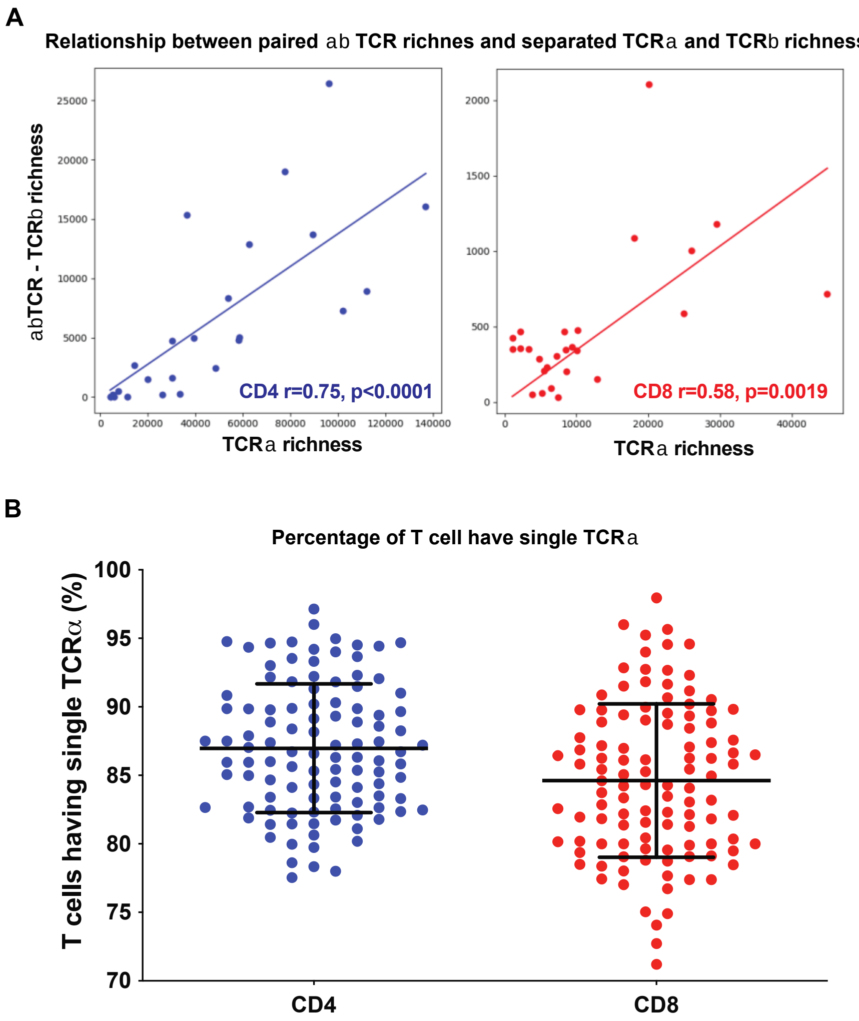

C

Equations for calculating paired ab TCR richness based on separated TCRa and TCRb richness

CD4 Paired ab TCR richness = 0.138 x [(0.870 x TCRa) + TCRb] + Max (0.870 x TCRa, TCRb)

CD8 Paired ab TCR richness = 0.035 x [(0.846 x TCRa) + TCRb] + Max (0.846 x TCRa, TCRb)

Figure 4: Development of equations to predict paired αβ TCR richness based on the unpaired TCRα and TCRβ sequences. A. Linear relationship between 1) the paired αβ TCR richnesses minus the maximum unpaired richnesses and 2) the sum of the unpaired richnesses, TCRα and TCRβ, from αβ TCR data of single cell analysis. 745,182 and 158,305 αβ TCRs from $CD4^+$ and $CD8^+$ T cells, respectively, were used in this analysis. B. Percentage of T cells with a single TCRα. The values are derived via the analyses of 108 and 111 single T cell samples for $CD4^+$ and $CD8^+$, respectively, using published data with secondary TCRα information (see method for details). C. Equations for calculating paired αβ TCR richness based on unpaired TCRα and TCRβ richness of $CD4^+$ and $CD8^+$ T cells. TCRα richness is preceded by a factor indicating average contribution to TCRα richness by primary TCRα's, compensating contributions to TCRα richness by secondary TCRα's in bulk-sequencing.
